# Design and Simulation of Broadband SiN Waveguide-Integrated GeSn Single-Photon Avalanche Detectors at Very-Near-Infrared to Telecommunication Wavelengths

**DOI:** 10.3390/s26082404

**Published:** 2026-04-14

**Authors:** Pawaphat Jaturaphagorn, Nattaporn Chattham, Apichart Pattanaporkratana, Papichaya Chaisakul

**Affiliations:** Department of Physics, Faculty of Science, Kasetsart University, Bangkok 10900, Thailand; pawaphat.ja@ku.th (P.J.); fscinpc@ku.ac.th (N.C.); fsciacp@ku.ac.th (A.P.)

**Keywords:** GeSn, single-photon avalanche detectors, Si-rich SiN waveguide

## Abstract

We investigate the potential to adopt waveguide-integrated GeSn single-photon avalanche detectors (SPADs) over a wideband wavelength range from very-near-infrared to telecommunication wavelengths based on an Si-rich SiN waveguide platform via an end-fire coupling approach. Electrical properties of GeSn SPAD heterodiodes are investigated, including their I–V characteristics, electric field distribution, charge sheet doping variation, avalanche triggering probabilities, dark count rate, and afterpulsing probability, to identify the appropriate critical parameters and to reliably benchmark against previous related simulation works. Notably, to enable a waveguide-integrated GeSn SPAD for the entire wavelength of interest, this paper finds that, among several potentially important parameters, the coupling efficiency between the input waveguide and the GeSn SPAD plays a very critical role in determining the single-photon detection efficiency (SPDE) performance, and a suitable GeSn absorber thickness should be carefully considered according to the chosen Sn content. Interestingly, although the coupling efficiency and SPDE are significantly varied between the longer wavelengths of 1310 and 1550 nm and the shorter wavelengths of 700 and 900 nm, an acceptable SPDE performance can be maintained for all wavelengths of interest for both close end-fire coupling (no gap between the amorphous Si-rich SiN waveguide and the GeSn SPAD) and a 50 nm gap assumption for simpler fabrication.

## 1. Introduction

Single-photon detection capability is crucial in several future and present technological applications such as quantum information and sensing [1], Light Detection and Ranging (LiDAR) [2], and medical imaging [3]. Several types of single-photon detectors (SPDs) are available, including photomultipliers [4], superconducting SPDs [5,6], and semiconductor single-photon avalanche photodiodes (SPADs) [7]. The photomultiplier option tends to suffer from a high dark count rate (DCR) and low single-photon detection efficiency (SPDE), while the superconducting SPDs require cryogenic temperatures to render relatively high SPDE with a low DCR. Currently, an SPAD is considered as one of the promising options to enable a single-photon detector with a sufficiently high SPDE and acceptable DCR without the requirement of cryogenic temperature [8]. Significantly, to potentially enable compact and low-cost quantum technologies compatible with high-volume Si-based manufacturing, on-chip waveguide-integrated single-photon detectors based on Group IV Si-compatible materials have received significant research attention [9,10,11,12,13,14,15,16]. For the superconducting SPDs option, waveguide-integrated superconducting nanowire single-photon detectors (SNSPDs) have shown strong SPDE and a low DCR, albeit at cryogenic temperatures [9,10,11]. On the other hand, waveguide-integrated Si-based SPADs have shown the potential to deliver usable values of SPDE and DCR in practical Peltier cooling environments or at room temperature [12,13,14,15,16,17]. Waveguide Ge-on-Si SPADs have been demonstrated at the near-infrared wavelength of 1310 nm [12,13] with an SPDE as high as 38% around 125 K [13]. Recently, surface-illuminated Ge-on-Si SPADs showed the possibility to operate at room temperature with an SPDE of 12% [18], and the possibility of waveguide integration has been theoretically discussed via 3D finite-difference time-domain (3D-FDTD) simulations [17]. On the contrary, Si SPADs have been shown to operate at a wavelength shorter than 1.1 µm via Monte Carlo simulation [14] and experimentally [15], with an SPDE of 35–45%, around a practical temperature of 250 K. Interestingly, increased incorporation of Sn content into Ge leads to a redshift of the energy bandgap in Ge-rich GeSn alloys, raising their optical absorption coefficient, which can significantly enhance the photodetection efficiency of GeSn-based detectors [19,20,21,22,23,24]. Q. Chen et al. [8] theoretically showed that cavity-enhanced surfaced-illuminated GeSn/Si SPADs could be expected to render an SPDE as high as 80% at room temperature around the optical wavelength of 1550 nm. Moreover, using the mode matching technique and finite element method (FEM) simulations, R. A. Soref et al. [16] demonstrated that GeSn/Si SPADs closely end-fire coupled with a Silicon-on-insulator (SOI) waveguide could render an SPDE of more than 30–80% at room temperature at the optical wavelengths of 1550 and 2000 nm thanks to the superior absorption properties of GeSn.

In this paper, we investigate the potential to adopt waveguide-integrated GeSn SPADs over a wideband wavelength range from 700 nm to 1550 nm (very-near-infrared to telecommunication wavelengths). The wavelength regions are important in the development of Ge-based optical detection [25]; moreover, the development of SPADs to support detection events from 700 nm to 1500 nm is instrumental in quantum information processing as the widely used telecommunication wavelength of 1550 nm can be employed to enable second harmonic generation at 775 nm for quantum state generation [26,27,28,29,30]. Section 2 describes the SPAD structure considered in this paper and indicates the scope of its key parameters, which we consider differently with respect to previous works. Section 3.1 investigates the electrical characteristics of the GeSn SPAD active region to verify our simulation works according to the available reported data and to identify a good GeSn SPAD design. Section 3.2 demonstrates the potential of waveguide-integrated GeSn SPADs for wideband application from very-near-infrared to telecommunication wavelengths, based on butt coupling with an Si-rich SiN waveguide, proposed in our previous investigation of a waveguide-integrated single-photon light source [31]. Section 4 provides the conclusions of and important remarks about this work.

## 2. Setup and Methods

**Figure 1:**
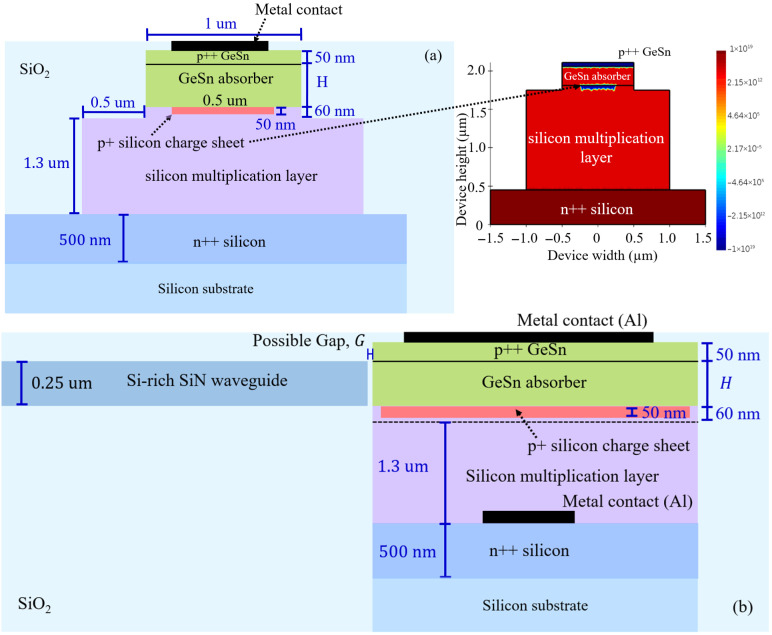
The schematics of (**a**) the SPAD device configuration, where the inset shows the carrier distribution with an Si charge sheet hole concentration of $5\times 10^{17}\;cm^{-3}$ and p++ GeSn and n++ Si layers with a doping level of $\sim 1\times 10^{19}\;{\mathrm{cm}}^{-3}$ (Ansys Lumerical Multiphysics). (**b**) Side view of the investigated optical coupling from a strip Si-rich SiN waveguide to the SPAD.

As shown in Figure 1a, an SPAD with the separate absorption and carrier multiplication (SACM) approach [8,13,16,32,33,34] is selected. The separation of the absorption and multiplication layers by a charge sheet has been shown to be efficient in generating sufficiently high electric fields in the multiplication layer for the avalanche effect, and the modest electric field in the absorption region is low enough to avoid band-to-band and trap-assisted tunneling but still high enough to allow drifting of photogenerated electrons from the absorption region to the multiplication one. Indeed, using finite element analysis, Ref. [13] presented the overall design rule of the SACM structure for a Ge-on-Si SPAD which will be adopted in this work. Accordingly, we choose the design that has the mesa etched into the multiplication layer just below the charge sheet region selectively ion-implanted in the Si, resulting in an electric field that is confined within the structure, and not at the mesa sidewall [13]. Therefore, the diameter of the mesa is less than the diameter of the multiplication layer by 1 µm. The metal top and bottom contacts can be formed with the p++ GeSn and n++ Si layers of the heterostructure, with a doping level of $\sim 1\times 10^{19}\;{\mathrm{cm}}^{-3}$, to be comparable with the other PIPIN SPAD structure [32]. Consistent with previous works [16], our preliminary investigation of the SPAD region indicates that the typical geometrical parameters of a SPAD of mesa diameter 1 µm, p++ GeSn layer thickness 50 nm, p+ Si charge sheet thickness 50 nm, multiplication region height 1.3 µm could be effectively employed. As shown in Figure 1b, to focus our investigation on the wideband very-near-infrared to telecommunication wavelengths waveguide integration of the GeSn SPAD, an Si-rich SiN waveguide is chosen as a waveguide material due to its wide transmission window, which covers the range from visible to mid-IR with a low extinction coefficient [35]. Moreover, we have previously shown that the Si-rich SiN waveguide platform could be used to support broadband optical coupling from single-photon sources in the relevant wavelength range [31]. Consistent with our previous investigation results in [31], the Si-rich SiN waveguide thickness is set to 0.25 µm. For the GeSn absorber, we consider three different Sn contents of GeSn, 3.6, 6.5, and 8.4%, from which their optical properties at room temperature could be approximated [20] over a wide spectral range. Electrical and bandgap properties are estimated as described in [36]. In our estimation, we found that, at 8% Sn, the GeSn energy bandgap turns from an indirect to a direct gap, which corresponds well with previous research [1,37], enabling our results to comprehensively cover both indirect and direct bandgap regions. In addition, although a close end-fire couple between an amorphous waveguide and semiconductor is practically possible [38] and has been adopted in previous studies [16], we also consider the effect of a possible gap (G) between the Si-rich SiN waveguide and the GeSn SPAD, as pointed out in other end-fire coupling works [39,40]. The simulations procedure is based on a Multiphysics approach. The carrier drift–diffusion equation is solved by the full-vectorial finite-difference method for electrical analysis of the SPAD, including the I–V and electric field distribution. In the meantime, for electro-optics simulation, Maxwell’s wave equations are consistently solved by the 3D-FDTD method together with the carrier drift–diffusion equation (Ansys-Lumerical 2025 R1: FDTD and Multiphysics software package), to define the SPAD under illuminating conditions with the smallest mesh size of ∼1 nm, and a perfectly matched layer (PML) is used in order to suppress reflections from the boundaries.

## 3. Results and Discussion

### 3.1. Electrical Properties of the Investigated GeSn SPADs

**Figure 2:**
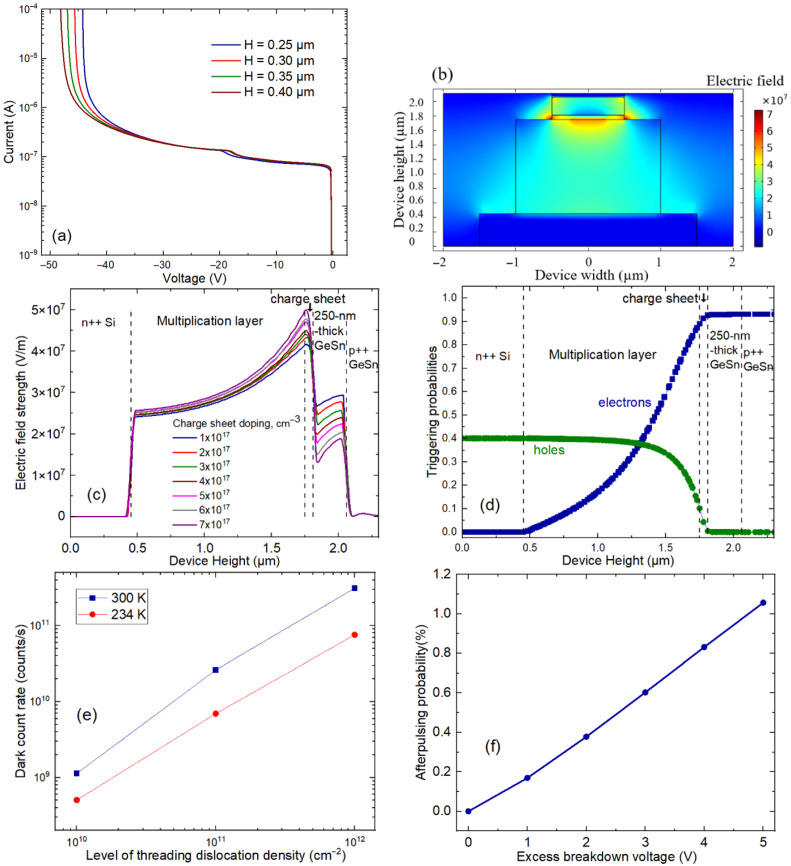
(**a**) SPAD I–V characteristics at different values of GeSn absorber thickness (H) with 8.4% Sn. (**b**) Spatial electric field distribution in the case of H = 0.25 µm. (**c**) Electric field strengths at different charge sheet doping variations from $1\times 10^{17}\;{cm}^{-3}$ to $7\times 10^{17}\;{cm}^{-3}$. (**d**) Electron and hold avalanche triggering probabilities along device vertical height at the center of the PIPIN SPAD structure. (**e**) DCR at each threading dislocation density at 5 V excess bias voltage and a GeSn absorber thickness of 250 nm. (**f**) Afterpulsing probability at a threading dislocation density of 10^12^ cm^−2^ at each excess bias voltage, in order to be both comparable and conservative with respect to [16].

Firstly, the electrical characteristics of the GeSn SPAD heterodiode depicted in Figure 1a are investigated with respect to the critical parameters of GeSn absorber thickness (H), p+ Si charge sheet doping level, threading dislocation density, and excess bias voltage. The SPAD I–V characteristics at different values of GeSn absorber thickness with 8.4% Sn are reported in Figure 2a. Calculations of electron and hole impact ionization coefficients at 300 K are consistent with [16] following the Okuto–Crowell model [41]. The I–V curves indicate the avalanche effect with a breakdown voltage (V_br_) estimated to be 42 V, 43 V, 44 V, and 45 V, defined as the voltage at which the current reaches 1 μA [32], for a GeSn absorber thickness of 250 nm, 300 nm, 350 nm, and 400 nm, respectively. The level of V_br_ increases with higher GeSn absorber thickness, consistent with the fact that higher values of bias voltage are required to achieve the electric field strength required for the avalanche effect. Figure 2b shows the obtained spatial distributions of the electric field for the case of 250 nm thick GeSn with 8.4% Sn. H = 250 nm is according to the lower breakdown voltage presented in Figure 2a, and the optimized coupling condition and SPDE values will be later discussed. Satisfactorily, a sufficiently high electric field region is achieved in the multiplication layer below the charge sheet to allow the avalanche effect, while a lower electric field (to avoid the tunneling effect [32]) still exists in the GeSn absorption region necessary for the drifting of photogenerated electrons toward the multiplication layer. The effect of the doping level in the p+ Si charge sheet is presented in Figure 2c. The hole concentration in the charge sheet layer is one of the key factors of the device because it directly affects the relative level of the electric field distribution between the absorption and multiplication regions. As the hole concentration in the Si charge sheet increases from $1\times 10^{17}\;cm^{-3}$ to $7\times 10^{17}cm^{-3}$, the electric fields in the GeSn absorption region gradually decrease while those in the multiplication one gradually increase. Specifically, at the charge sheet doping level $7\times 10^{17}cm^{-3}$ in our structure, a modest electric field of $1–2\times 10^{7}$ V/m comparable to that in [16] can be maintained in the GeSn absorption region, and the electric field of >$5\times 10^{7}$ V/m is obtained in the multiplication layer below the charge sheet, which is consistent with the preferred electric field levels in the previous work. It should be noted that an electric field of more than $7\times 10^{7}$ V/m should be also avoided in the multiplication layer to make the band-to-band tunneling effect negligible [16]. We calculate avalanche triggering probabilities by following the numerical methods proposed by R. McIntyre [42,43] and also recently adopted in [16] (Equation (10)): $\partial P_{e}(x,y)/\partial y=\left(1-P_{e}\right)\alpha_{e}\left(E\right)\left(P_{e}+P_{h}-P_{e}P_{h}\right)$ and $\partial P_{h}(x,y)/\partial y=-\left(1-P_{h}\right)\alpha_{h}\left(E\right)\left(P_{e}+P_{h}-P_{e}P_{h}\right)$, in which $P_{e}$ and $P_{h}$ are the avalanche triggering probability for electrons and holes, the subscriptions e and h stand for n- and p-type materials, and α represents the impact ionization coefficient according to the Okuto–Crowell model [41]. x and y represent the position along the device width and height direction, as indicated in the inset of Figure 1a. Figure 2d shows electron and hold avalanche triggering probabilities at 5 V excess bias voltage (Geiger mode) along the center of the PIPIN SPAD structure. Consistent with [8,44,45], the maximum electron avalanche trigger probability is obtained in the top GeSn layer and decreases to zero at the n++ Si layers, while the hole avalanche trigger probability is zero in the top GeSn layer and increases to attain its maximum value at the n++ Si layer. The DCR, evaluated according to the procedure described in [46] (Equation (14)), $DCR=L\int_{x}\int_{y}(P_{e}+P_{h}-P_{e}P_{h})G\left(x,y\right)dxdy$, where L is the SPAD length, depends directly on the avalanche triggering probabilities and generation rate of charge carriers per unit volume G(x,y), which is based subsequently on the carrier lifetime [47] (Equation (21)), the energy difference between the recombination center and the intrinsic Fermi energy level, and the electric field strength. The carrier lifetime is defined as $\tau =1/\left(\sigma v_{th}N_{D}N_{TD}\right)$, where σ is the trap cross-section, $v_{th}$ is the carrier thermal velocity, $N_{D}$ is the threading dislocation density, and $N_{TD}$ is the number of traps per unit length of dislocation, with employed values consistent with [16]. Figure 2e shows that the DCR of the GeSn SPAD increases as the level of the threading dislocation density and the operating temperature increase. At a threading dislocation density of 10^12^ cm^−2^ and 300 K, a DCR of ~10^11^ counts/s is obtained, which is still comparable to [8,16] for sensing and optical quantum applications, indicating the reliability of the electrical characteristics obtained from our calculation. One of the key important metrics for an avalanche diode is the afterpulsing probability (AP) [46] (Equation (21)), which is due to the fact that, when the avalanche pulse is triggered by the stream of carriers, it will always be followed by a pulse that is caused by detrapping electrons. $AP=\int_{0}^{W}\alpha_{e}\left(y\right)\left[\int_{y_{1}}^{y_{2}}N_{tni}\cdot \left(1-f_{tni}\right)\cdot n\cdot \sigma \cdot \eta \cdot P_{e}\left(y\right)\right]dy$, where $N_{tni},\;n,\;\eta ,\;and\;\left(1-f_{tni}\right)$ are the electron trap density of the ith level, electrons generated in an avalanche pulse, electron emission probability, and electron fraction of unoccupied traps, respectively [46]. The values are integrated along the height of the device and within the width of the avalanche region. Consistent with [16,46], we choose the electron lifetime of the first trap to be 10 ns, to be conservative compared to [16], and the energy difference between the electron first trap (second trap) and the intrinsic Fermi level is set at 0.20 eV (0.15 eV) within the scope of both references. Figure 2f shows the afterpulsing probability at a threading dislocation density of 10^12^ cm^−2^ for a GeSn (8.4% Sn content) absorber thickness of 250 nm at different excess bias voltages. As expected, the afterpulsing probability increases directly with the level of excess bias voltage. The level of the afterpulsing probability of the GeSn SPAD of 1% is comparable with that obtained from Silicon at room temperature [46], reaffirming the potential of the investigated GeSn SPAD.

### 3.2. Si-Rich SiN Waveguide-Integrated Wideband GeSn SPAD

**Figure 3:**
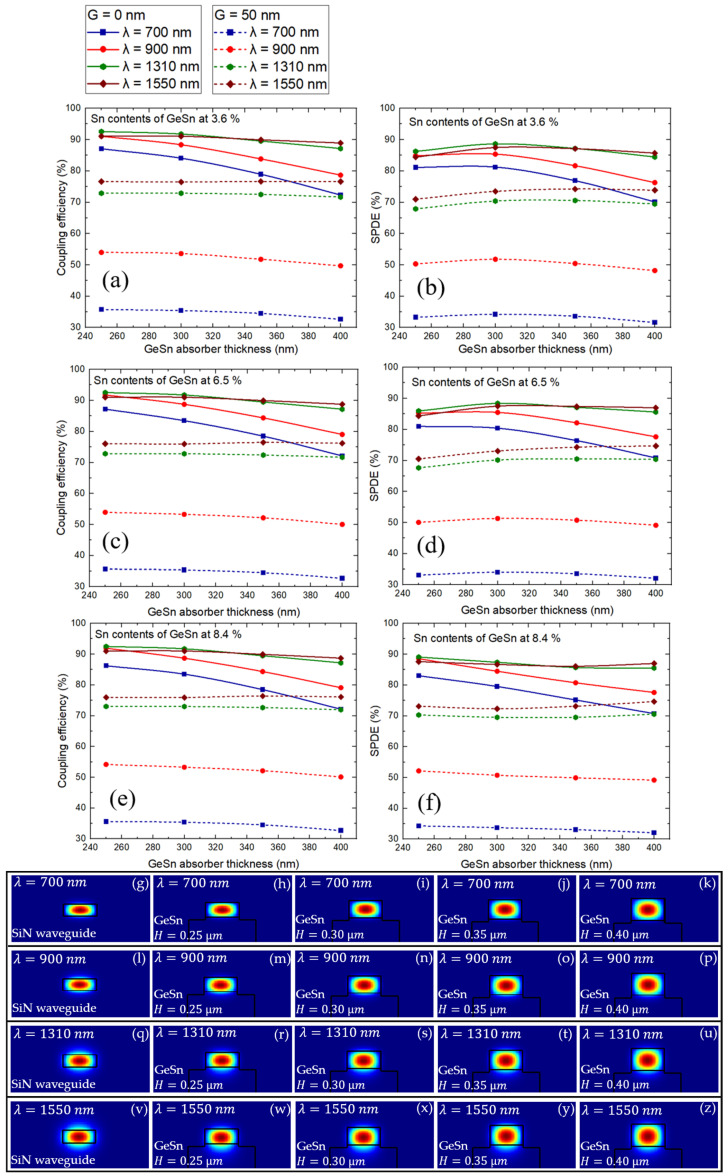
Coupling efficiency between the Si-rich SiN waveguide at Sn contents of (**a**) 3.6%, (**c**) 6.5%, and (**e**) 8.4% and the SPAD. Single-photon detection efficiency (SPDE) performance at Sn contents of (**b**) 3.6%, (**d**) 6.5%, and (**f**) 8.4% for wideband application from the very-near-infrared to the telecommunication wavelengths considering either a close end-fire coupling or a 50 nm gap between the Si-rich SiN waveguide and the GeSn SPAD. (**g**–**z**) Electric field distribution of optical modes from Si-rich SiN input waveguides and GeSn SPADs at all optical wavelengths of interest.

To holistically investigate the potential of the Si-rich SiN waveguide-integrated GeSn SPAD for wideband application from the very-near-infrared to the telecommunication wavelengths, the coupling efficiency between the Si-rich SiN waveguide and the SPAD and the single-photon detection efficiency (SPDE) performance are investigated over the four wavelengths of 700 nm, 900 nm, 1310 nm, and 1550 nm, covering all wavelength regions of interest, as shown in Figure 3. As discussed in [8,16], the SPDE is basically dependent on the coupling efficiency ($\eta_{coup}$), the probability of a detectable avalanche ($\eta_{ava}$), the collection efficiency of the photogenerated carriers from the absorber to the multiplication layer ($\eta_{inj}$), and the absorption efficiency of the absorber ($\eta_{abs}$). $\eta_{inj}$ should not be a limiting factor to the SPDE as the electric field in the GeSn absorption layer is strong enough to accelerate electrons toward the multiplication layer. $\eta_{ava}$ is obtained by solving avalanche triggering equations [16] (Equation (10)). $\eta_{abs}$ is directly related to the material absorption coefficient (α), optical confinement factor (γ), and device length (L) as $\eta_{abs}=1-e^{-\alpha \gamma L}$. As a result, the coupling efficiency ($\eta_{coup}$) could be the critical parameter in determining the SPDE performance of the waveguide-integrated SPAD, because, in these works, $\eta_{abs}$ is sufficiently large across all wavelengths of interest and Sn concentrations [20] and $\eta_{ava}$ is directly proportional to the excess bias voltage above the breakdown, chosen to be 5 V according to Figure 3f.

From Figure 3a,c,e, we can see that, when a close end-fire coupling is employed (G = 0), as in [16], the coupling efficiency of the fundamental quasi-transverse electric (quasi-TE) mode from the Si-rich SiN to the GeSn region remains higher than 85% using a relatively thin GeSn absorber thickness (H) of 250 nm for all Sn contents of GeSn at (a) 3.6%, (c) 6.5%, and (e) 8.4%, respectively. Interestingly, the coupling efficiency surprisingly decreases as H becomes thicker from 250 to 400 nm. This can be attributed to the optimized optical mode matching between the GeSn absorber and that of the Si-rich SiN waveguide, the thickness of which is set to be 250 nm as mentioned before. Moreover, we find that the coupling efficiency is evidently higher at the longer wavelengths of 1310 nm and 1550 nm than at the shorter wavelengths of 700 nm and 900 nm. This is due to the fact that shorter wavelengths lead to higher optical confinement in both the Si-rich SiN waveguide and GeSn structure; therefore, the effective index difference of light in the two different materials is larger. Figure 3b,d,f shows the obtained SPDE over the relevant optical wavelengths for all Sn contents of GeSn at (b) 3.6%, (d) 6.5%, and (f) 8.4%, respectively. Indeed, SPDE exhibits the same trend as the coupling efficiency for every optical wavelength and Sn content. This clearly validates our assumption that the coupling efficiency is the critical parameter in determining the SPDE performance of the waveguide-integrated SPAD. It is important to note that, for Sn contents of (b) 3.6% and (d) 6.5%, the SPDE values at a GeSn absorber thickness of 300 nm are slightly higher than that at 250 nm. This is because a thicker absorber region will increase the absorption efficiency of the absorber; therefore, the SPDE can be slightly improved using a GeSn absorber thickness of 300 nm despite a lower coupling efficiency than that at 250 nm. Notably, at the highest Sn content of (f) 8.4%, we see no improvement of the SPDE at the thicker GeSn absorber thickness of 300 nm, because the absorption coefficient at such a high Sn concentration [20] is significantly larger than that at a lower Sn concentration; therefore, a thicker GeSn absorber does not play an important role in improving the SPDE. In addition, Figure 3g–z reports the electric field distribution of optical modes from Si-rich SiN input waveguides and GeSn SPADs at all optical wavelengths of interest. Consistent with the above explanation, the optical modes of the GeSn absorber with lower values of thickness H are more comparable to those of the Si-rich SiN waveguides for all optical wavelengths, supporting our finding that a thinner GeSn absorber thickness of 250 nm results in a better SPDE performance than that of 400 nm. It is important to note that GeSn’s much higher refractive index compared with Si makes GeSn thickness the parameter that dominates the optical mode profile, thereby significantly influencing the optical coupling performance and the SPDE.

Interestingly, although a close end-fire couple with an amorphous waveguide is practically possible [38], the possible gap (G) between the Si-rich SiN waveguide and the GeSn SPAD would be useful to lower fabrication complexity. From Figure 3a,c,e, we can see that the introduction of G ~50 nm adversely affects the coupling efficiency at the shorter wavelength region of 700 and 900 nm much more than at the longer wavelengths of 1310 nm and 1550 nm. This is because a longer wavelength will be less affected by the possible gap than a short wavelength due to the smaller relative size of the gap with respect to the longer wavelength cases. Fortunately, with G ~50 nm, at the shortest wavelength of 700 nm, SPDE is still above the projected acceptable value of 30% for waveguide-integrated SPADs for quantum communication applications [16]. According to Table 1, compared with the obtained SPDE from different waveguide-coupled SPADs on Si reported in the literature, the GeSn-on-Si SPAD closely end-fire coupled with an Si-rich SiN waveguide proposed in this work can potentially render a competitive SPDE over wideband wavelengths from the very-near-infrared to telecommunication wavelengths. It is important to note that our work reports a slightly higher value of SPDE than [16] because we consider various cases of relatively low and high Sn concentration ranging from 3.6% to 8.4%.

**Figure 4:**
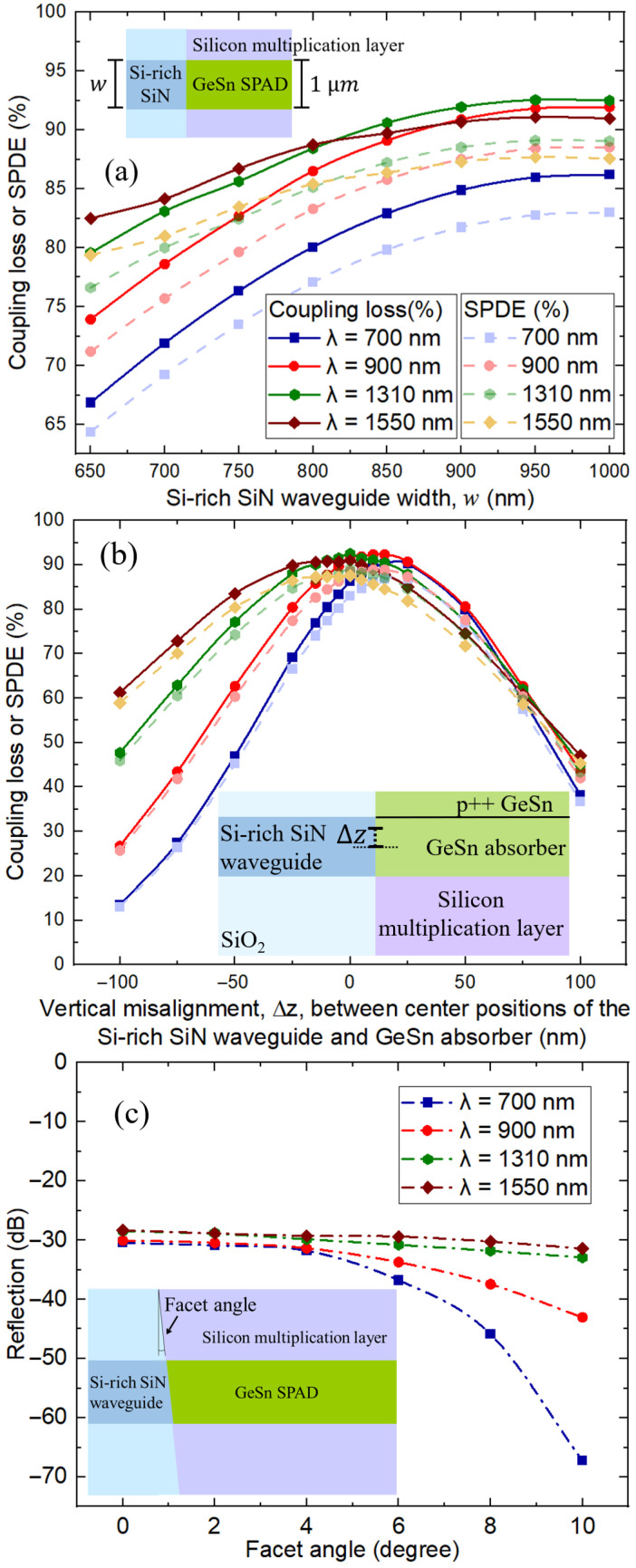
(**a**) Coupling efficiency and SPDE at different Si-rich SiN waveguide width (w) values while the width of the waveguide-integrated GeSn (8.4% Sn content) SPAD is kept at 1 µm. (**b**) The coupling efficiency and SPDE with respect to the vertical misalignment (∆z) between the center position of the Si-rich SiN waveguide and the GeSn absorber part of the GeSn SPAD. (**c**) Back reflection between the Si-rich SiN waveguide and the GeSn SPAD interface at different facet angles.

As the thickness of the Si-rich SiN waveguide is set to be 250 nm, consistent with our previous investigation results [31], it is worth investigating the effect of Si-rich SiN waveguide width variation to assess the effect of input waveguide dimensional shift on the SPAD performance. Figure 4a reports the coupling efficiency and SPDE at different Si-rich SiN waveguide width (w) values while the width of waveguide-integrated GeSn (8.4% Sn content) SPAD is kept at 1 µm. Both coupling efficiency and SPDE progressively decrease as w is reduced from 1 µm for all optical wavelengths of interest. Therefore, the widths of the Si-rich SiN input waveguide and the GeSn SPAD should be chosen to be the same over the entire wavelength, which also facilitates a simpler fabrication alignment process. This affirms our statement in the previous section studying the effect of GeSn absorber thickness: that optical mode matching between the GeSn absorber and the Si-rich SiN waveguide is critical for the coupling efficiency, which is the main parameter in determining the SPDE performance of a waveguide-integrated SPAD. Practically, it is critical to consider the effect of the vertical misalignment between the center position of the Si-rich SiN waveguides and the GeSn absorber part of the GeSn SPADs. Figure 4b reports the coupling efficiency and SPDE with respect to the vertical misalignment (∆z). As expected, the performance at the longer wavelengths of 1310 and 1550 nm is less affected by the vertical misalignment comparing to that at the shorter wavelengths of 700 and 900 nm due to the larger mode size of the former. Promisingly, we find that both the coupling efficiency and SPDE can be competitively maintained if the misalignment in vertical position is within ±25 nm for all optical wavelengths of interest, which is consistent with the typical requirement found in our previous works [48,49,50] and continued progress in advancing CMOS processes toward large-scale integration of sub-wavelength photonic structures on a Si platform [51,52]. Importantly, back reflection should be also verified to ensure seamless transfer of light between different materials [40,50,53] as a low back reflection is required in Si-based photonic integrated-circuit optical coupling (<−28 dB) [54]. Figure 4c shows the values of the back reflection between the Si-rich SiN waveguide and GeSn SPAD interface at different facet angles. A back reflection value of ∼−28 dB can be achieved with a facet angle of 0° at every wavelength of interest. As expected, at a higher facet angle, the back reflection decreases; nevertheless, a facet angle of 0° with <−28 dB reflection is preferable as increasing facet angles will unavoidably lead to higher optical coupling loss [55].

## 4. Conclusions and Remarks

We investigated the potential to adopt waveguide-integrated GeSn SPADs over a wideband wavelength range from 700 nm to 1550 nm (very near-infrared to telecommunication wavelengths), which could be instrumental in the development of quantum information technologies. Initially, we described the SPAD structure considered in this paper and investigated its electrical properties, including its I–V characteristics, electric field distribution, charge sheet doping variation, avalanche triggering probabilities, DCR, and afterpulsing probability. The investigation allowed us to both identify the appropriate critical parameters of GeSn absorber thickness (H) and charge sheet doping level and benchmark our electrical simulation results against previous related simulation works to reaffirm the reliability of the projected characteristics of the investigated SPAD. Subsequently, we investigated the potential of the Si-rich SiN waveguide-integrated GeSn SPAD over the four wavelengths 700 nm, 900 nm, 1310 nm, and 1550 nm, covering the very-near-infrared to telecommunication wavelengths. This paper found the following: (1) The coupling efficiency between the input waveguide and the GeSn SPAD plays a very critical role in determining the SPDE performance over the entire wavelength of interest. (2) When trying to efficiently enable good wideband operation over the entire wavelength of interest, a thicker GeSn absorber does not always lead to a higher SPDE performance. A thinner GeSn absorber thickness of 250 nm results in a better SPDE performance than one of 400 nm. (3) Different Sn content in the GeSn absorber leads to a slight difference in optimized absorber thickness. A waveguide-integrated SPDE with lower Sn contents of 3.6% and 6.5% requires a thicker absorber than one with a higher Sn content of 8.4% to achieve optimized SPDE. That is, GeSn with lower Sn contents has a lower absorption coefficient; therefore, thicker absorber layers help improve the SPDE despite having lower coupling efficiency. GeSn with 8.4% Sn content already attains a direct gap absorption characteristic with a significantly higher absorption coefficient, so a thicker GeSn absorber does not significantly improve the SPDE. (4) The coupling efficiency is significantly varied between the longer wavelengths of 1310 and 1550 nm and the shorter wavelengths of 700 and 900 nm; the higher optical confinement of the latter leads to a larger effective index mismatch between different materials and a higher coupling loss. Moreover, the coupling efficiency at the shorter wavelength region is much more adversely affected by the possible gap (G) between the Si-rich SiN input waveguide and the GeSn SPAD than at the longer wavelengths, due to the smaller relative size of the gap with respect to the longer wavelength cases. Interestingly, SPDE is still above the projected acceptable values of 30% with G ~50 nm at the shortest wavelength of 700 nm. (5) The width of the Si-rich SiN input waveguide and the GeSn SPAD can be chosen to be the same for optimized coupling efficiency and SPDE over the entire wavelength, which is also significant in terms of facilitating a simpler fabrication alignment process. (6) A close end-fire coupling between the Si-rich SiN waveguide and the SPAD device can be employed with a low level of back reflection even with a facet angle of 0° at every wavelength of interest. Via 3-D finite-difference time-domain techniques together with electrical simulation, this work contributes toward a holistic understanding of the potential and limitations of Si-rich SiN waveguide-integrated GeSn SPADs over a wideband wavelength range from the very-near-infrared to telecommunication wavelengths, which could be critical in several applications in future chip-scaled quantum communication systems.

## Figures and Tables

**Table 1:** Comparison of SPDE from different waveguide-coupled SPADs on Si reported in the literature at comparable voltage.

Waveguide-Coupled SPAD on Si	Wavelength	Operating Voltage (V)	Temperature	SPDE
[12] Ge-on-Si coupled with SOI waveguide (*experimental, 2017*)	1310 nm (Telecom)	~32–37 V	80 K	5.27%
[14] P-N junction Si waveguide coupled with Si_3_N_4_ waveguide (*theoretical, 2018*)	640 nm (Visible)	~20–50 V	243 K	45%
[15] P-N junction Si waveguide coupled with SiN waveguide (*experimental, 2024*)	~500 nm (Visible)	~20 V	Room temperature	>6%
[16] GeSn-on-Si SPADs coupled with SOI waveguide (*theoretical, 2019*)	1550 and 2000 nm (Telecom and mid-IR)	~48 V	Room temperature	30–80%
GeSn-on-Si SPADs coupled with Si-rich SiN waveguide (*theoretical, this work*)	700–1550 nm (very-near-infrared to telecom)	~47 V	Room temperature	30–90%

## Data Availability

The data presented in this study are available upon request from the corresponding authors.
